# Transient efficacy of buparvaquone against the US isolate of *Theileria orientalis* Ikeda genotype in sub-clinically infected cattle

**DOI:** 10.3389/fvets.2024.1421710

**Published:** 2024-07-26

**Authors:** Reginaldo G. Bastos, Amany Hassan, Cynthia K. Onzere, David R. Herndon, Nicolas F. Villarino, Jacob M. Laughery, Lindsay M. Fry

**Affiliations:** ^1^Animal Disease Research Unit, USDA-ARS, Pullman, WA, United States; ^2^Department of Veterinary Microbiology and Pathology, Washington State University, Pullman, WA, United States; ^3^Department of Animal Medicine, Faculty of Veterinary Medicine, University of Alexandria, Alexandria, Egypt; ^4^Department of Veterinary Clinical Sciences, College of Veterinary Medicine, Washington State University, Pullman, WA, United States

**Keywords:** *Theileria orientalis*, Ikeda genotype, buparvaquone, cattle, anti-*Theileria* therapeutic, *Theileria* subclinical infection

## Abstract

**Introduction:**

*Theileria orientalis*, an economically significant tick-borne hemoparasite, infects cattle globally. The *T. orientalis* Ikeda genotype, transmitted by *Haemaphysalis longicornis* ticks, is associated with clinical manifestations characterized by anemia, abortions, and mortality, although subclinical infections prevail. Despite the common occurrence of subclinical infections, therapeutic interventions targeting *T. orientalis* Ikeda in such cases are currently lacking, impeding effective parasite control measures. To address this critical knowledge gap, we assessed the efficacy of buparvaquone (BPQ) in eliminating the *T. orientalis* Ikeda, US isolate, in sub-clinically infected cattle.

**Methods:**

Twelve sub-clinically infected calves, identified by the presence of *T. orientalis* in peripheral blood alongside the absence of fever and anemia, were enrolled in the study. Six calves received two treatments of the BPQ label dose (2.5 mg/kg) at a 48-h interval, while additional three calves received the drug at a dosage of 6 mg/kg following the same regimen. Three untreated calves served as controls.

**Results and discussion:**

Endpoint and quantitative PCR analyses revealed that BPQ exerted a transient effect on *T. orientalis* parasitemia. Parasites remained undetectable in peripheral blood until weeks 4 and 11 post-treatment in animals administered 2.5 mg/kg and 6 mg/kg of BPQ, respectively. Intriguingly, following recrudescence, administering 6 mg/kg to animals previously treated with 2.5 mg/kg did not result in a reduction in parasite load. Pharmacokinetic analysis data suggested that escalating the dosage led to a less than proportional increase in serum concentrations of BPQ. Moreover, a significant yet reversible decrease (*p* < 0.05) in blood urea nitrogen was observed in animals treated with the drug, irrespective of the dosage. Despite parasitemia relapse, animals treated with 6 mg/kg BPQ exhibited a noteworthy decrease (*p* < 0.05) in IgG levels specific to the *T. orientalis* major piroplasm surface protein compared to controls and animals treated with 2.5 mg/kg of the drug.

**Conclusion:**

BPQ did not demonstrate efficacy in clearing subclinical *T. orientalis* Ikeda infection. Future investigations are warranted to explore innovative therapeutic modalities that, in synergy with vaccines and diagnostic assays, can facilitate the development of comprehensive programs aimed at controlling and eradicating this parasite.

## 1 Introduction

*Theileria orientalis* is an apicomplexan tick-borne hemoprotozoan parasite that causes an economically important disease in cattle globally (1–4). Phylogenetic studies based on the *T. orientalis* major piroplasm surface protein (MPSP) and small subunit rRNA genes have identified 11 different genotypes. Among these genotypes, Ikeda, Chitose, and Buffeli are the most prevalent in Japan, Australia, and New Zealand (1, 5, 6). It has been demonstrated that infection with the Ikeda genotype, also known as genotype 2, is generally associated with clinical disease characterized by hemolytic anemia, fever, jaundice, prostration, abortion, and death (7–9). *T. orientalis* outbreaks caused by Ikeda have been recently reported in the US, posing a serious threat to the country’s cattle industry (3). The Ikeda outbreaks in the US have coincided with the emergence of *Haemaphysalis longicornis* ticks in the country. *H. longicornis* is the primary tick vector for the transmission of *T. orientalis* worldwide (10), and our group recently demonstrated the competence of this tick species as a biological vector in the transmission of the *T. orientalis* Ikeda genotype, US isolate (11).

Unlike *Theileria parva* and *Theileria annulata*, the causative agents of East Cost fever and tropical theileriosis, respectively, the asexual reproduction of *T. orientalis* schizonts in leukocytes of the mammalian host does not induce uncontrolled cell proliferation (1, 12, 13). In this regard, *T. orientalis* is like *Theileria equi*, a non-transforming *Theileria* species and one of the parasites responsible for equine piroplasmosis (14, 15). Clinical disease caused by *T. orientalis* Ikeda is associated with the asexual replication of merozoite/piroplasm stages of the parasite in the host red blood cells (RBC), leading to hemolytic anemia and decrease in packed cell volume (PCV) (1). Although infection with *T. orientalis* Ikeda often causes mild anemia, the disease may become severe and occasionally fatal due to co-infections and stress associated with animal management (16, 17). Despite the mild clinical symptoms associated with acute *T. orientalis* Ikeda infection, significant losses in production have been reported in infected animals (1, 18–21). Considering all these factors, development of effective control measures against *T. orientalis* Ikeda are urgently needed.

Generally, control of tick-borne hemoparasites relies on strategies to decrease tick infestation, and the use of vaccines and anti-parasitic therapeutics. The use of acaricides is unsustainable as it can select for resistant tick populations and cause toxicity to animals and the environment (22). No vaccines and therapeutics are currently available for the control of *T. orientalis*. Collectively, these gaps pose a serious threat to the cattle industry in affected areas, particular in countries, such as the US, where the parasite is emerging and mirroring the distribution of its major tick vector *H. longicornis* (3). Thus, development of vaccines and identification of effective therapeutics against *T. orientalis* Ikeda are essential in the control of the parasite and its subsequent eradication.

Buparvaquone (BPQ) is a hydroxynaphthoquinone antiprotozoal drug, like parvaquone and atovaquone, originally developed to control *Plasmodium* species, which has a broad-spectrum effect against apicomplexan parasites (23, 24). The drug has been extensively used, at the label dose of 2.5 mg/kg, to control *T. annulata* and *T. parva*. Administration of BPQ efficiently reduces the load of *T. annulata* and *T. parva* during acute infection, allowing infected cattle to gain time to mount a protective immune response and survive acute disease (23, 25–28). It has been also demonstrated that BPQ is effective against *T. orientalis* if the treatment is implemented during the earlier stages of the infection (29, 30). However, conclusive evidence of the efficacy of BPQ, used at the label and higher doses, in clearing *T. orientalis* Ikeda in chronically infected cattle remains unknown. Therefore, this study aimed to evaluate the efficacy of BPQ in the clearance of *T. orientalis* Ikeda, US isolate, in sub-clinically infected cattle. Results demonstrate that BPQ has a theilericidal effect on Ikeda; however, relapse of parasitemia was observed in peripheral blood of all experimental animals. Data on pharmacokinetics of BPQ as well as the effect of the drug on blood cell count and chemistry, and serology for *T. orientalis* MPSP were also evaluated in this study.

## 2 Materials and methods

### 2.1 Cattle

A total of 12 spleen-intact Jersey-Charolais crossed calves, 6 to 12 months of age, were used in this study. All animal study procedures described in this investigation were approved by the Washington State University Institutional Animal Care and Use Committee (IACUC# 6981).

### 2.2 Subclinical infection of *Theileria orientalis* Ikeda, US isolate, in cattle

Calves were inoculated with either blood stabilate or *H. longicornis* salivary gland (SG) infected with *T. orientalis* Ikeda, USA isolate. Blood stabilate was produced as previously described (11). Production of SG stabilate was performed following a similar protocol previously described for *T. parva* (31). Parasite load in blood and SG stabilates was assessed by qPCR, as described below, and ranged from 5.5×10^6^ to 3.8×10^5^ parasites per mL, respectively. Animals were inoculated intravenously with blood stabilate (5–10 mL inoculum) or subcutaneously with SG stabilate (1 mL inoculum). After infection, animals were monitored for alterations in temperature, PCV, number of blood leukocytes and RBC. For this study, subclinical infection of *T. orientalis* Ikeda, US isolate, was defined by the presence of parasite DNA in peripheral blood with the concomitant absence of fever and anemia.

### 2.3 Detection of *Theileria orientalis* Ikeda DNA in bovine peripheral blood

Detection and quantification of *T. orientalis* Ikeda DNA in peripheral blood of infected calves were performed by endpoint PCR and quantitative real-time PCR (qPCR), respectively, targeting the MPSP gene (AP011946.1). Genomic DNA (gDNA) was extracted from 100 uL of EDTA-containing whole blood from the infected animals using the QIAamp DNA Mini Kit (Qiagen), according to the manufacturer’s protocol. The following minor modifications were made: samples were incubated in lysis buffer and proteinase K for 30 min at 56°C; and elution was performed with 50 uL of pre-warmed (56°C) buffer AE and repeated for a final extraction volume of 100 uL. *T. orientalis* was detected in DNA from whole blood by endpoint PCR targeting a 776-bp segment of the MPSP gene using specific primers (forward 5’ctttgcctaggatacttcct 3′ and reverse 5′ acggcaagtggtgagaact 3′) (3, 11). Reactions consisted of 22.5 uL of Accuprime Pfx Supermix (Thermo Fisher Scientific, Waltham, MA, USA), 140 nM final concentration of each primer, and 2 uL of DNA template. Amplification was carried out in the C1000 thermal cycler (Bio-Rad, Hercules, CA, USA) with an initial denaturation of 95°C for 5 min and 35 cycles of 95°C for 15 s, 57°C for 15 s, and 68°C for 1 min. Amplicons were visualized via agarose gel electrophoresis. DNA sequencing of PCR products was carried out for amplicon confirmation. qPCR was performed on gDNA extracted from whole blood as described above, targeting a 113-bp portion of the MPSP gene from *T. orientalis* Ikeda. Reactions consisted of 1x SsoFast Evagreen™ Supermix (Bio-Rad), 200 nM final concentration of specific primers (forward 5′ ccttcggactacaagcctc 3′ and reverse 5′ tgtgagactcaatgcgccta 3′), and 2 uL of template DNA. The *T. orientalis* amplicon was cloned into pCR™4Blunt-TOPO™ (Thermo Fisher Scientific) to construct a standard curve using serial 10-fold dilutions (10^6^ to 10^1^) of plasmid DNA for absolute quantification. qPCR was performed using the CFX Opus Real-Time PCR System (Bio-Rad) with an initial denaturation 98°C for 2 min followed 40 cycles of 95°C for 5 s and 58°C for 5 s. Efficiency of amplification and melt curve analyses were performed to evaluate analytical sensitivity and specificity, respectively, of the MPSP qPCR. Quantification was reported as the number of copies of MPSP per 1 μL of blood.

### 2.4 BPQ treatment

BPQ (Buparvex Injection, Biomeda Inc., Dublin, Ireland) was used in this study. The drug was administrated intramuscularly (IM), following the manufacture’s recommendation. Twelve sub-clinically *T. orientalis* infected calves were randomly distributed in three groups. In group 1, six calves were treated with the recommended BPQ label dose of 2.5 mg/kg. In group 2, three calves were treated with 6 mg/kg of BPQ. In both groups 1 and 2, BPQ treatment occurred at approximately 6–8 weeks post *T. orientalis* infection (sub-clinical phase of the infection) with two injections, 48 h apart. Group 3 (three calves) remained untreated and served as controls.

### 2.5 Analytical method for pharmacokinetics of BPQ

Evaluation of the BPQ concentration in serum samples was performed by pharmacokinetics analysis in two and three calves treated with 2.5 mg/kg or 6 mg/kg, respectively. Briefly, BPQ serum analysis was conducted using reverse phase high performance liquid chromatography method, as previously described (32, 33). The analysis was conducted with a 2,695 separations module, and a 2,487 UV absorbance detector (Waters, Milford, MA, USA). A XBridge C18 (4.6 × 100 mm, 3.5 μm) column was used for the separation. Ammonium acetate (0.02 M, pH 3.6) and acetonitrile were used as the mobile phase. The absorbance was measured at 251 nm and the flow rate was 1 mL/min. Samples that were previously frozen were thawed at room temperature, mixed, and 100 μL of serum was transferred to a 13 × 100 mm screw top tube followed by 15 μL of lovastatin (internal standard, 100 μg/mL), and 1 mL ether. The mixture was vortexed for 60 s and then centrifuged for 20 min at 1000 *x*g. The organic layer was transferred to a glass tube and evaporated to dryness with nitrogen gas. Samples were reconstituted in 250 μL of mobile phase and 100 μL was analyzed. A typical standard curve for the analysis was prepared by fortifying untreated, pooled serum with BPQ, which produced a linear concentration range of 0.025–100 μg/mL. Average recovery for both BPQ and the internal standard was 100%. The intra-and inter-assay variability ranged from 1.5 to 8.8%, and the lower limit of quantification was 0.01 μg/mL.

### 2.6 Hematological analysis

Cell blood count was evaluated using the ProCyte One™ Hematology Analyzer (IDEXX Laboratories, Inc., Westbrook, ME, USA), following the manufacturer’s protocol. Peripheral blood was collected into Vacutainer® tubes containing EDTA (BD Company, Franklin Lakes, NJ, USA) at several timepoints post-BPQ treatment. After collection, whole blood samples were homogenized for 5 min and the numbers of total leukocytes, lymphocytes, monocytes, neutrophils, and RBC were measured. White blood cell populations were counted as 1,000 cells/mL of blood, and RBC were counted as 1,000,000 cells/mL of blood. Blood chemistry was evaluated using the Catalyst One Veterinary Blood Chemistry Analyzer (IDEXX Laboratories), following the manufacturer’s protocol. The following parameters were analyzed in the chemistry panel: glucose, creatinine, blood urea nitrogen (BUN), BUN-creatinine ratio, phosphorus, calcium, total protein, albumin, globulin, albumin-globulin ratio, alanine transaminase (ALT), alkaline phosphatase (ALP), gamma-glutamyl transferase (GGT), total bilirubin, and cholesterol. The IDEXX VetConnect PLUS software (IDEXX Laboratories) was used for visualization of data from the ProCyte DX™ and the Catalyst One Veterinary Blood Chemistry Analyzer.

### 2.7 MPSP ELISA

The level of antibodies against the *T. orientalis* Ikeda MPSP in BPQ-treated and control calves was evaluated by indirect ELISA (iELISA), using recombinant MPSP (recMPSP) as the antigen. Briefly, for antigen production, the *T. orientalis* Ikeda MPSP gene (AP011946.1) was codon optimized for the expression in prokaryotic cells, synthesized as a 6-His tag fusion gene, and cloned into pET30a (GenScript, Piscataway, NJ, USA). *Escherichia coli* BL21 Star™ (DE3) (GenScript) were transformed with the recombinant plasmid and selected in LB medium containing kanamycin (50 ug/ml). Culture was incubated at 37°C at 200 rpm, and once cell density reached to OD = 0.6–0.8 at 600 nm, 0.5 mM IPTG was added to induce expression. Protein expression was monitored by immunoblot using an anti-6-His tag monoclonal antibody (GenScript). The recombinant protein was purified by nickel column, using standard protocols. Serum samples from experimental animals were obtained by standard protocols at several timepoints post-BPQ treatment. For the MPSP iELISA, 96-well Immulon™ 2HB microtiter plates (Thermo Fisher Scientific) were coated overnight at 4°C with 50 μL of recMPSP (2 μg/mL) in 1× Coating Buffer (BioLegend, San Diego, CA, USA). After that, excess of antigen was removed, and the plates were blocked with 200 μL/well of Blocker™ Casein in PBS (Thermo Fisher Scientific) at room temperature (RT) for 1 h. Following the blocking step, serum samples were diluted 1/50 in 0.05% (v/v) Tween-20 in PBS (PBS-T), and 50 μL were added to duplicate individual wells. Plates were incubated at RT for 1 h, and after three washes in PBS-T, 50 μL of a 1/1000 dilution of anti-bovine IgG peroxidase-labeled secondary antibodies (SeraCare, Milford, MA, USA) were added to individual wells. Plates were then incubated at RT for 1 h. After that, plates were washed three times in PBS-T, two times in PBS, and developed with 55 μL of 1-Step™ Ultra TMB-ELISA Substrate Solution (Thermo Fisher Scientific). After 10-min incubation in the dark, the enzymatic reaction was stopped by adding 55 μL of TMB Stop Solution (0.2 M H_2_SO_4_) (SeraCare) to each well. Plates were read at optical density (OD) 450 nm using the Synergy HTX ELISA plate reader (BioTek, Winooski, VT, USA). Results are presented as the average of OD 450 nm values. A cutoff was determined by the average of negative samples (uninfected cattle sera; *n* = 10) plus three standard deviations.

### 2.8 Statistical analysis

Comparisons of temperature, numbers of RBC and leukocytes, PCV values, values of blood chemistry, and levels of anti-MPSP IgG between BPQ-treated and control animals were performed by the Mann–Whitney test, and a *p* value <0.05 was considered statistically significant. GraphPad Prism software version 9 (GraphPad Software, San Diego, CA) was used for all the statistical analyses. For pharmacokinetics analysis, the area under the serum concentration versus time profile (AUC) of BPQ was calculated using the trapezoidal rule, as previously described (34). Dose proportionality was evaluated for both dose levels by calculating the dose-normalized AUC from 0 to 48 h after the first drug administration (AUC0-48 h) and from 0 to 168 h (after the second drug administration) (AUC0-168 h) and peak of drug concentration (Cmax) (Cmax/dose) (35).

## 3 Results

### 3.1 BPQ has a transient theilericidal effect against *Theileria orientalis* Ikeda, US isolate, in sub-clinically infected calves

**Figure 1:**
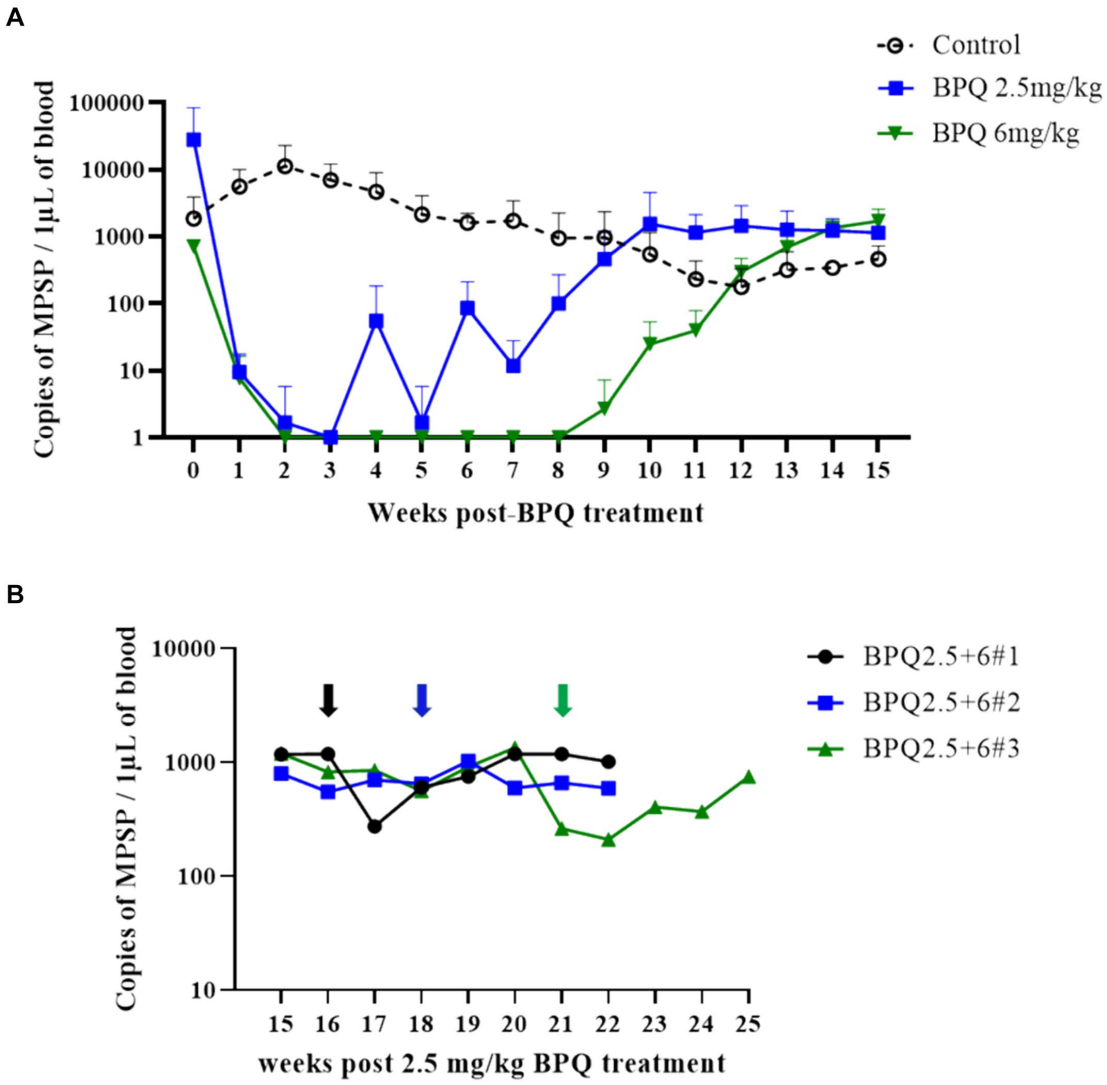
Quantification of *Theileria orientalis* Ikeda, US isolate, DNA in peripheral blood of cattle treated with buparvaquone (BPQ). **(A)** Animals treated with 2.5 mg/kg (*n* = 6) (blue line), 6 mg/kg (*n* = 3) (green line), and untreated controls (*n* = 3) (black line). **(B)** Animals (*n* = 3) treated with 2.5 mg/kg BPQ following additional administration of 6 mg/kg of the drug. Black, blue and green arrows in panel B indicate the timepoint that the respective three animals were retreated with 6 mg/kg BPQ.

To evaluate the effect of BPQ on the parasitemia of *T. orientalis* Ikeda genotype, US isolate, in sub-clinically infected cattle, animals were treated with 2.5 mg/kg or 6 mg/kg of the drug, and the parasite load after treatment was monitored by endpoint PCR and qPCR. Results of endpoint PCR demonstrated that *T. orientalis* was undetectable in peripheral blood of calves that received 2.5 mg/kg of BPQ from 3 to 8 weeks post-treatment. After that period, parasite was consistently detected (Table 1). Considering the resurgence of parasites in these treated animals, a second round of two treatments with a 48-h interval and comprising 6 mg/kg of BPQ, was conducted. Interestingly, endpoint PCR data showed that this second, higher dose treatment had no effect on the parasite, which was continuously detected in blood of retreated animals (Table 1). Results of endpoint PCR demonstrated undetectable levels of parasite in peripheral blood of animals that received 6 mg/kg of BPQ for 9 to 11 weeks after treatment (Table 1). However, parasite recrudescence was also observed in this high-dose BPQ-treated group (Table 1). *T. orientalis* DNA was continuously detected by endpoint PCR in the control, untreated calves throughout the experiment (20 weeks) (Table 1). Results of qPCR demonstrated >100-fold decrease in parasite load at week 1 post-treatment in both 2.5 mg/kg and 6 mg/kg treatment groups (Figure 1A). Parasite load fell below detectable levels or was marginally detected by qPCR in both treatment groups two weeks after the administration of BPQ. Initial parasite relapse (approx.100 parasites/1 μL of blood) in animals treated with 2.5 mg/kg was observed between 4 and 6 weeks post-BPQ treatment. Parasites were then consistently detected in this group of animals throughout the experiment. Interestingly, no quantifiable parasites were detected from weeks 2 to 8 in animals treated with 6 mg/kg of the drug. However, parasite relapse in these animals was initially observed by qPCR 9 weeks post-treatment, after which they were consistently detected throughout the experiment (Figure 1A). Peak of parasite recrudescence (>1,000 parasites/1 μL of blood) in animals that received 2.5 mg/kg or 6 mg/kg occurred at weeks 10 and 15, respectively (Figure 1A). To address the effect of dose and retreatment on parasite load, we performed qPCR on blood samples from the three calves that were re-treated with 6 mg/kg of BPQ after the initial treatment with 2.5 mg/kg of the drug. Interestingly, the second round of treatment with a higher dose of BPQ had no observable effect on the level of parasites in peripheral blood of the previously treated animals, confirming the endpoint PCR data (Table 1; Figure 1B). Collectively, results demonstrated that BPQ had a transient effect on the load of *T. orientalis* Ikeda, US isolate, in sub-clinically infected calves (Table 1; Figure 1). Parasite recrudescence was observed in all experimental animals, and administration of 6 mg/kg to animals that previously received the label dose of BPQ did not significantly decrease *T. orientalis* load in peripheral blood. In addition, no significant differences in parasite load were observed after recrudescence by comparing BPQ-treated and control calves (Figure 1).

**Table 1:** Efficacy of BPQ (2.5 mg/kg and 6 mg/kg) against *Theileria orientalis* Ikeda, US isolate, in sub-clinically infected cattle.

WPBPQ	C#1	C#2	C#3	2.5#1	2.5#2	2.5#3	2.5 + 6#1	2.5 + 6#2	2.5 + 6#3	6#1	6#2	6#3
wk0	+	+	+	+	+	+	+	+	+	+	+	+
wk1	+	+	+	−	−	−	−	−	−	−	−	−
Wk2	+	+	+	−	−	−	−	−	−	−	−	−
Wk3	+	+	+	−	−	−	−	−	−	−	−	−
Wk4	+	+	+	−	−	+	−	−	−	−	−	−
Wk5	+	+	+	+	−	−	−	−	−	−	−	−
Wk6	+	+	+	+	−	+	−	−	−	−	−	−
Wk7	+	+	+	−	−	−	+	−	−	−	−	−
Wk8	+	+	+	+	+	−	+	+	−	−	−	−
Wk9	+	+	+	+	+	−	+	+	+	−	−	−
wk10	+	+	+	+	−	+	+	+	+	+	−	−
wk11	+	+	+	+	+	+	+	+	+	−	−	−
wk12	+	+	+	+	+	+	+	+	+	+	+	+
wk13	+	+	+	+	+	+	+	+	+	+	+	+
wk14	+	+	+	+	+	+	+	+	+	+	+	+
wk15	+	+	+	+	+	+	+	+	+	+	+	+
wk16	+	+	+	+	+	+	+	+	+	+	+	+
wk17	+	+	+	+	+	+	+	+	+	+	+	+
wk18	+	+	+	+	+	+	+	+	+	+	+	+
wk19	+	+	+	+	+	+	+	+	+	+	+	+
wk20	+	+	+	+	+	+	+	+	+	+	+	+
wk21	ND	ND	ND	ND	ND	ND	+	+	+	ND	ND	ND
wk22	ND	ND	ND	ND	ND	ND	+	+	+	ND	ND	ND
wk23	ND	ND	ND	ND	ND	ND	+	+	+	ND	ND	ND
wk24	ND	ND	ND	ND	ND	ND	+	+	+	ND	ND	ND
wk25	ND	ND	ND	ND	ND	ND	+	+	+	ND	ND	ND
wk26	ND	ND	ND	ND	ND	ND	+	+	+	ND	ND	ND

WPBPQ, weeks post-buparvaquone treatment; BPQ, buparvaquone; C#, control animals with no BPQ treatment; 2.5#, animals treated with 2.5 mg/kg BPQ; 2.5 + 6#, animals treated with 2.5 mg/kg followed by 6 mg/kg BPQ. Grey cells indicate the week that animals received the second treatment dose of 6 mg/kg BPQ; 6#, animals treated with 6 mg/kg BPQ; ND, not done.

### 3.2 Relapse of *Theileria orientalis* Ikeda, US isolate, in BPQ-treated cattle is associated with increased temperature and decreased RBC and PCV

**Figure 2:**
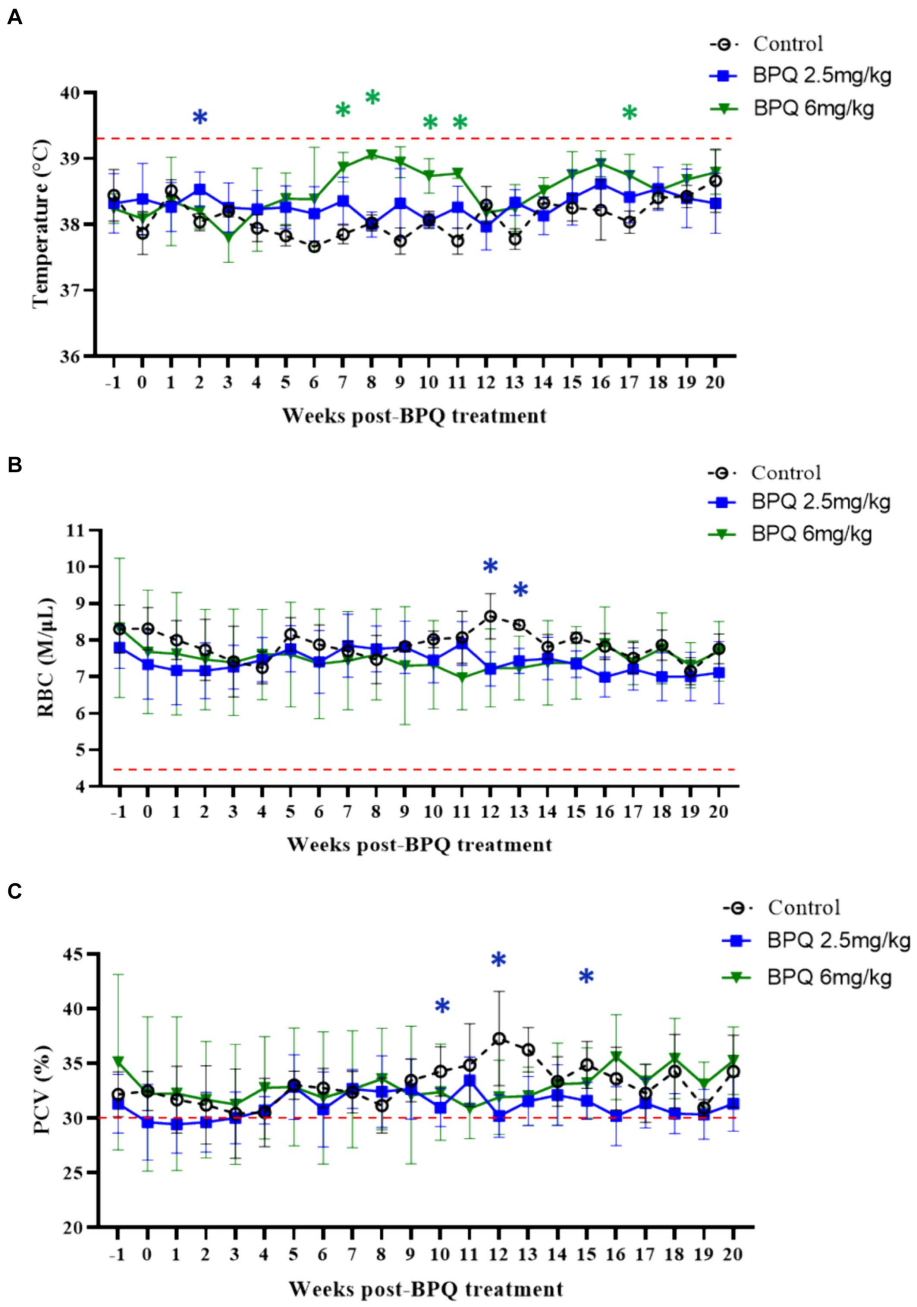
Temperature **(A)**, red blood cell (RBC) count **(B)**, and packed cell volume (PCV) **(C)** during buparvaquone (BPQ) treatment following relapse of *T. orientalis* Ikeda, US isolate. Animals treated with 2.5 mg/kg (*n* = 6) (blue line), 6 mg/kg (*n* = 3) (green line), and untreated controls (*n* = 3). Temperature results are shown as degrees Celsus while RBC and PCV are presented as million cells per micro litter of blood and percentage, respectively. Red lines in panels **(A–C)** indicate the threshold for fever (39.4°C), the physiological level for RBC (4.47 M/μL), and the physiological level for PCV (30%), respectively.

Next, we examined temperature, RBC numbers, PCV, and numbers of blood leukocytes during treatment followed by parasite relapse. Despite the presence of *T. orientalis* Ikeda DNA in peripheral blood of all calves used in this experiment at the initial BPQ treatment, animals had no fever and no alterations in the numbers of RBC and PCV (Figure 2). These results provide evidence that the experimental calves were at the sub-clinical phase of the infection during the drug treatment. Although it was not technically considered fever (≥39.4°C), data showed a significant (*p* < 0.05) increase in temperature in animals that received 2.5 mg/kg BPQ two weeks after the initial treatment. Similarly, animals treated with 6 mg/kg BPQ showed significant (*p* < 0.05) elevation in temperature between 6 to 11 weeks post-treatment (Figure 2A). Interestingly, in both experimental groups, increased temperature preceded the relapse of parasites in peripheral blood (Table 1; Figure 1A). A significant (*p* < 0.05) decrease in the numbers of RBC was also observed in calves treated with 2.5 mg/kg BPQ at 12 and 13 weeks post-treatment (Figure 2B). No significant alterations in RBC were detected in animals treated with 6 mg/kg BPQ. Significant (*p* < 0.05) drops in PCV were observed at weeks 10, 12 and 16 post-BPQ treatment in animals that received 2.5 mg/kg of the drug (Figure 2C). In contrast, no significant alterations in RBC were detected in animals treated with 6 mg/kg BPQ. Furthermore, no significant alterations were observed in the numbers of total leucocytes, lymphocytes, monocytes, and neutrophils in peripheral blood of the sub-clinically *T. orientalis*-infected animals treated with BPQ (Supplementary Table S1). These results demonstrated that relapse of *T. orientalis* Ikeda, US isolate, after BPQ treatment occurs concomitantly with a mild, but significant increase in temperature, and a decrease in RBC and PCV in sub-clinically infected cattle.

### 3.3 Effect of BPQ treatment on the antibody response to *Theileria orientalis* Ikeda MPSP

**Figure 3:**
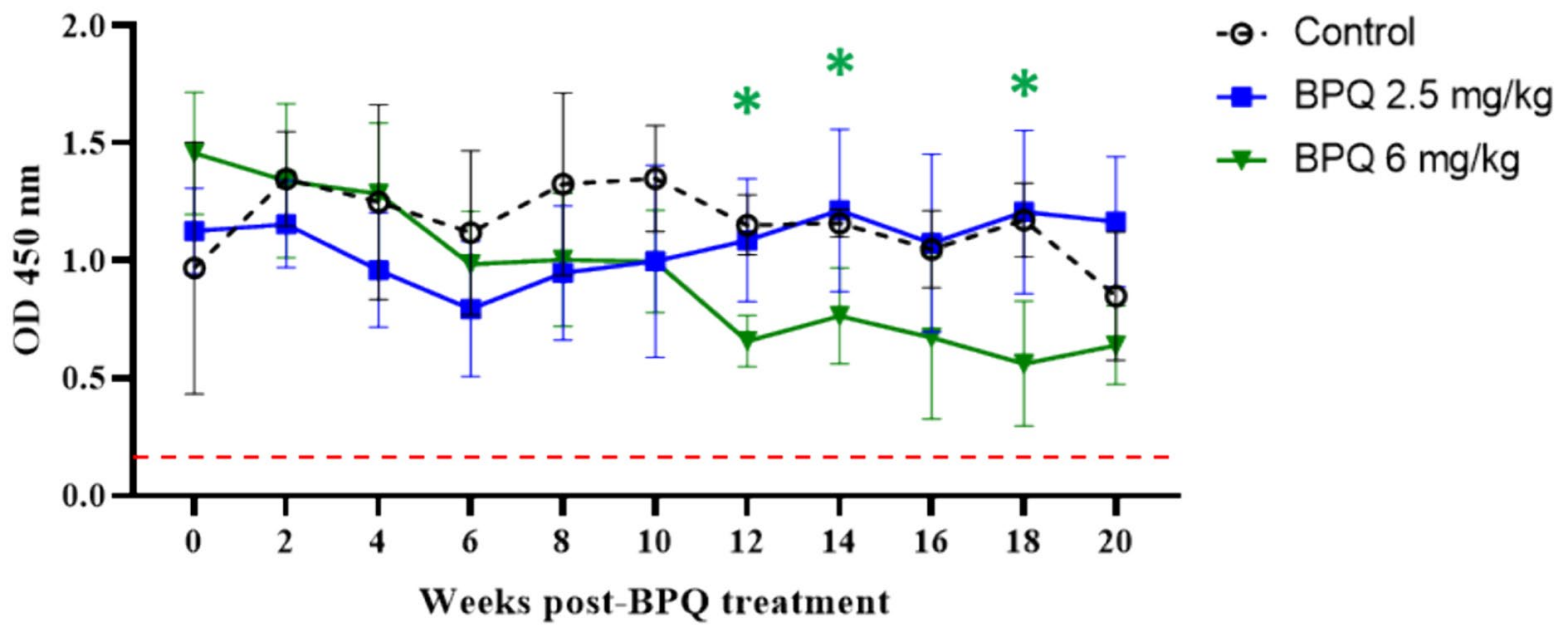
MPSP ELISA data of *T. orientalis* Ikeda, US isolate, infected cattle treated with 2.5 mg/kg (*n* = 6) or 6 mg/kg (*n* = 3) buparvaquone (BPQ), and control group (*n* = 3). Results are presented as the mean OD450 nm. Dashed line indicates the cutoff of 0.24 OD450 nm determined by the average of negative samples (uninfected cattle sera) plus three standard deviations. Green asterisks represent significant differences (*p* < 0.05) in the levels of anti-MPSP IgG in calves treated with 6 mg/kg PBQ compared to control untreated animals.

After showing a robust, but transient parasiticidal effect of BPQ against *T. orientalis* Ikeda in sub-clinically infected cattle, we investigated whether the reduction of parasite load induced by the drug treatment had any impact on the level of antibodies to *T. orientalis* MPSP. No significant differences were observed between animals that received 2.5 mg/kg of the drug and the control group, despite the tendency of decreased MPSP antibody levels around day 6 post-treatment (Figure 3). In contrast, data indicated a significant decrease (*p* < 0.05) in anti-MPSP IgG levels at 12, 14 and 16 weeks post-BPQ treatment in animals that received 6 mg/kg of the drug compared to controls (Figure 3). Regardless of the fluctuations of anti-MPSP antibodies, all calves, including BPQ-treated and control animals, showed significant levels of anti-MPSP IgG throughout the experiment. Collectively, despite the transient theilericidal effect of BPQ treatment in infected cattle, which resulted in undetectable levels of parasite in peripheral blood and a significant decrease in the levels of anti-IgG MPSP in animals treated with 6 mg/kg of the drug, all animals in this experiment were consistently positive for MPSP serology throughout the study (Figure 3).

### 3.4 BPQ pharmacokinetics and blood chemistry analysis

**Figure 4:**
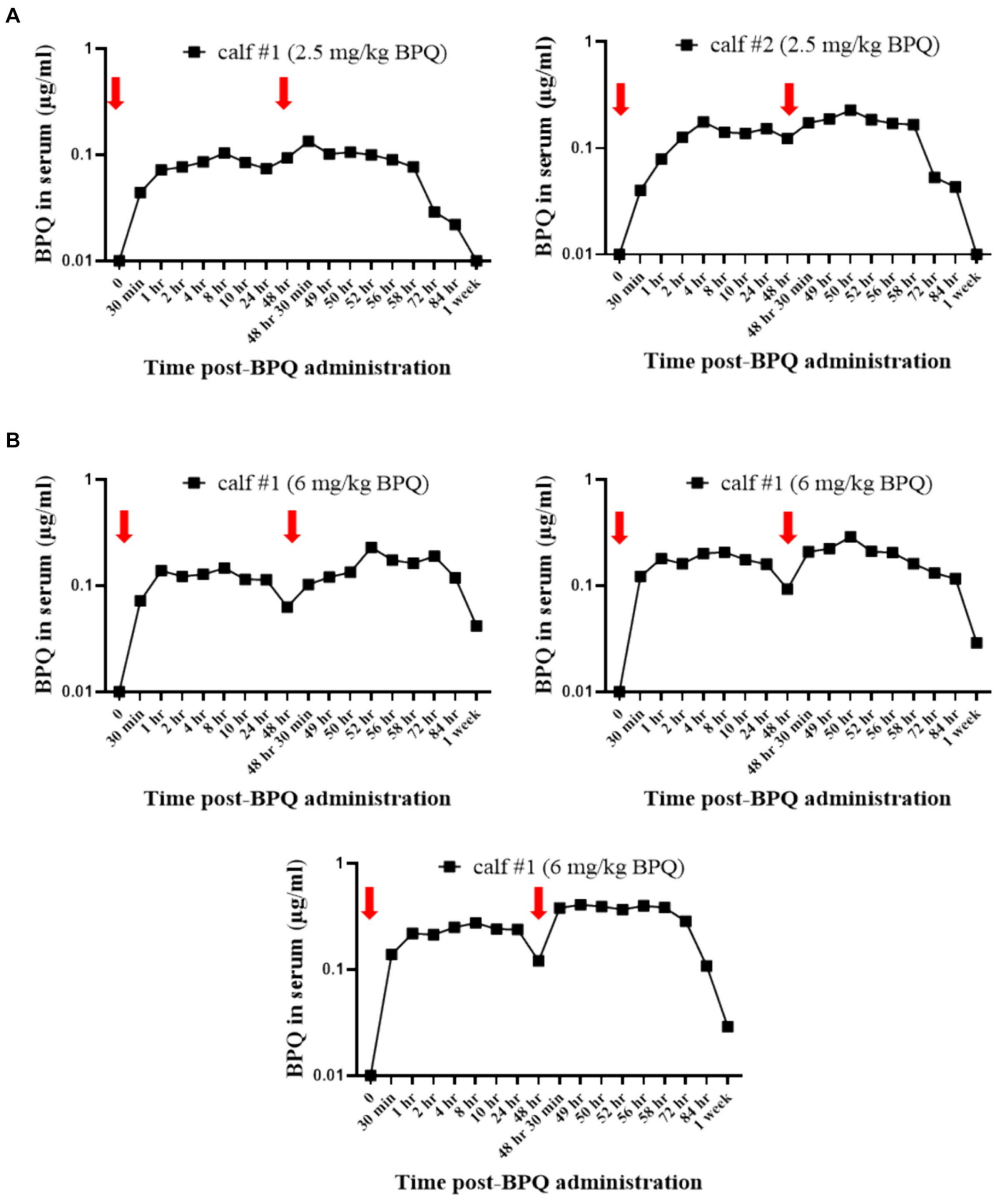
Pharmacokinetics analysis showing the concentration (μg/mL) of buparvaquone (BPQ) in sera of calves treated with 2.5 mg/kg **(A)** or 6 mg/kg **(B)**. Red arrows indicate the timepoints for drug administration.

**Figure 5:**
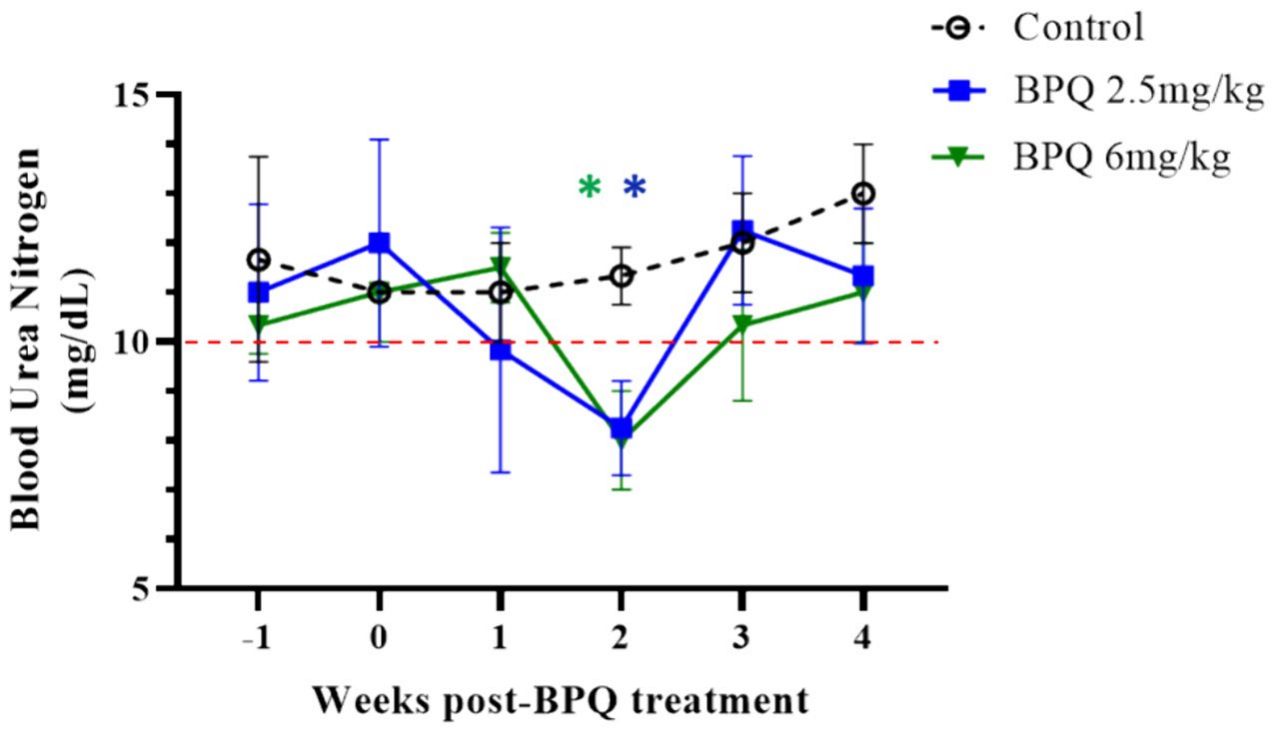
Levels of blood urea nitrogen (BUN) during buparvaquone (BPQ) treatment with 2.5 mg/kg (BPQ 2.5 mg/kg) (*n* = 6) or 6 mg/kg (BPQ 6 mg/kg) (*n* = 3), and controls (*n* = 3). Asterisks indicate *p* < 0.05 by comparing BPQ-treated groups versus control animals.

Considering the effect of BPQ on the parasitemia of *T. orientalis* Ikeda in sub-clinically infected cattle, it was of interest to investigate the kinetics of the drug after IM administration of 2.5 mg/kg or 6 mg/kg (Figure 4). Results of pharmacokinetics analysis showed that BPQ was detected in serum samples as early as 30 min after the first administration of the drug, with mean levels of 0.040 (±0) μg/mL and 0.112 (±0.034) μg/mL in calves that received 2.5 mg/kg or 6 mg/kg, respectively. The maximal concentrations were observed between 1 and 8 h post-drug administration. The concentration versus time profile revealed that once the maximal serum concentration was attained, the drug concentration was sustained for about 48 h, when it started to decline. After the first dose, AUC0-48 h ranged from 4 to 6.7 μg *h/mL and 7.5 to 16 μg*h/mL for the 2.5 mg/kg and 6 mg/Kg dose groups, respectively. After the second dose, the AUC0-168 h ranged from 3.9 to 7.4 μg*h/mL and 5.1 to 21 μg*h/mL for the 2.5 mg/kg and 6 mg/Kg dose groups, respectively. The Cmax after the first and second doses ranged from 0.11 to 0.13 μg/mL and 0.17 to 0.22 μg/mL for the 2.5 mg/kg dose group. The Cmax after the first and second doses ranged from 0.21 to 0.27 μg/mL and 0.13 to 0.39 μg/mL for the 6 mg/Kg dose group, respectively. A tendency of higher concentrations was observed in animals that received 6 mg/kg starting at 2 h after the first administration compared to the ones administered with 2.5 mg/kg. However, no statistical analysis was performed considering that only two and three animals treated with 2.5 mg/kg or 6 mg/Kg BPQ, respectively, were examined. The mean (AUC0-48 h /6 mg/kg)/(AUC0-48 h /2.5 mg/kg) was 0.59. The mean (AUC0-168 h /6 mg/kg)/(AUC0-168 h/2.5 mg/kg) was 1.31 μg/mL. The mean (Cmax/6 mg/kg)/(Cmax/2.5 mg/kg) was 1.48 and 1.43 μg/mL after the first and second doses, respectively. Considering that 6 mg/kg corresponds to 2.4 times more compound that 2.5 mg/kg, the results suggest a deviation from dose proportionality (Figure 4). Next, we examined a panel of chemistry metabolites in peripheral blood to investigate potential toxicity associated with the BPQ administration. Levels of BUN were significantly (*p* < 0.05) decreased in animals that received either 2.5 mg/kg or 6 mg/kg of BPQ two weeks after the administration of the drug compared to control animals (Figure 5). In fact, the levels of BUN at 2 weeks after BPQ treatment were lower than the normal reference interval. In contrast, considering BPQ-treated and control animals, no significant alterations were observed on the levels of glucose, creatinine, BUN-creatinine ratio, phosphorus, calcium, total protein, albumin, globulin, albumin-globulin ratio, ALT, ALP, total bilirubin, and cholesterol (data not shown). Altogether, data demonstrated that IM administration of BPQ resulted in slow release of the drug to peripheral blood of calves, with a tendency of higher concentrations in serum of animals that received 6 mg/kg than calves treated with 2.5 mg/kg. Also, BUN levels showed a significant but reversible decrease in all treated animals, regardless of the drug dose (Figures 4, 5).

## 4 Discussion

Availability of therapeutics that are able to clear infection in sub-clinically infected mammalian hosts is critical in the control and eradication of arthropod-borne infectious diseases, such as malaria, babesiosis, and theileriosis. *T. orientalis* is an economically important apicomplexan tick-borne parasite that poses significant challenges to the cattle industry in several countries (1–4). Infection with *T. orientalis* genotype Ikeda is generally associated with the development of fever and anemia during the initial phase of parasite replication, and this can have both short-and long-term impact on animal production (18). The Ikeda genotype is endemic in Japan, Australia, and New Zealand, and it is currently considered an emerging tick-borne parasite in the US (3). The absence of vaccines and effective therapeutics against *T. orientalis* Ikeda poses additional threats to the cattle industry, and this is of special concern in areas where the parasite has recently emerged, such as the US, and endemic balance has yet to be established. There are currently no drugs registered to treat cattle infected with *T. orientalis* Ikeda in the US, and only supportive therapy is available. Therefore, the goal of this study was to evaluate the efficacy of BPQ, as a monotherapeutic, to clear the parasite in cattle sub-clinically infected with *T. orientalis* Ikeda, US isolate. Our overall results demonstrated that BPQ has a robust theilericidal effect against the parasite. By evaluating the 2.5 mg/kg label dose and a higher dose of 6 mg/kg, we showed that parasite load fell to undetectable or marginal levels as early as 1 week after starting the treatment, corroborating a previous observation (36). Endpoint and qPCR approaches demonstrated that the parasite remained undetectable for up to 4 and 11 weeks after treatment with 2.5 mg/kg and 6 mg/kg of the drug, respectively. After that period, parasite relapse was observed in all treated animals, indicating that, despite the remarkable effect of BPQ on reducing parasite load, the drug was not effective in the clearance of the *T. orientalis* Ikeda, US isolate, in sub-clinically infected cattle.

The *T. orientalis* Ikeda genotype is of primary concern since it is associated with outbreaks of clinical disease and carrier subclinical infection. Upon infection with Ikeda, cattle develop a peak of parasitemia that may be accompanied by fever, hemolytic anemia, reduced PCV, jaundice and even death. However, most animals overcome this acute phase of the infection and become asymptomatic, lifelong carriers of the parasite (1, 4, 37). Therefore, the presence of sub-clinically infected animals is common in endemic areas (2, 4, 38). Re-emergence of clinical signs in chronically infected cattle may occur with recrudescence of parasitemia due to stress associated with animal management (1, 2, 39). In addition, the presence of sub-clinically infected animals in areas where *T. orientalis* tick vectors, especially *H. longicornis*, are present represents an additional risk of spreading the disease to naïve cattle herds (11). In this study, animals were at the subclinical phase of the infection (approximately 6 to 8 weeks after the parasite inoculation), as demonstrated by the absence of fever and anemia, and the concomitant presence of parasites in peripheral blood (7.1×10^2^ to 2.8×10^4^ parasites per μL of blood). Collectively, the present results are in agreement with previous data on the chronicity aspects associated with *T. orientalis* Ikeda (4). Interestingly, we demonstrated an elevation of temperature and drop in RBC count and PCV following the relapse of parasitemia in BPQ-treated animals, which supports previous observation on the potential recrudescence of clinical disease in chronically infected animals (2). In addition, the results add relevant information on the pattern of antibody response to *T. orientalis* Ikeda MPSP in sub-clinically infected cattle, which complements previous studies (40). We observed a significant decrease in the levels of anti-MPSP IgG in animals treated with 6 mg/kg BPQ, despite the noticeable parasite relapse. It was beyond the scope of this study to investigate the correlation between parasite load and antibody response to *T. orientalis* Ikeda MPSP, and additional investigations are necessary. Therefore, further studies are needed to define this correlation and other aspects, such as the usefulness of serological assays based on the *T. orientalis* Ikeda MPSP to identify acute and chronically infected cattle.

Hydroxynaphthoquines, such as atovaquone and parvaquone, are quinolone derivative compounds that inhibit the electron transport chain in the mitochondria of apicomplexan parasites. These drugs were originally developed as anti-malaria therapeutics, and then they showed to have a broad effect against numerous protozoal parasites (23). As a next generation of hydronaphthoquine, BPQ has shown to be several times more effective than atovaquone and parvaquone against *Theileria*, *Eimeria*, and *Plasmodium* parasites (23, 41). Therefore, BPQ is recommended to be used at lower doses than atovaquone and parvaquone to induce similar outcomes with reduced toxicity (42). BPQ is currently the drug of choice for controlling lymphoproliferative species of *Theileria*, such as *T. parva* and *T. annulata*. It has been shown that administration of BPQ during the early stages of infection with *T. parva* and *T. annulata* significantly reduces the levels of parasitemia, mitigating lymphoproliferation, which allows the infected animals to mount a protective immune response and survive acute infection (41, 43–47). Studies have also shown that BPQ is effective against non-transforming *Theileria* spp. (48–51). Efficacy studies using BPQ in splenectomized calves demonstrated that the drug is effective against the *T. orientalis* Ikeda genotype (30). Administration of the label dose 2.5 mg/kg early during acute infection induced a significant reduction in *T. orientalis* parasitemia. In fact, this previous study showed that no parasites were detected in BPQ-treated animals by day 6–7 after the drug administration (30). Our results support these previous data, showing that BPQ was effective at rapidly reducing the parasitemia to undetectable levels in treated animals as early as one week after treatment (50, 51). In this study, we further investigated whether BPQ was able to clear *T. orientalis* Ikeda in sub-clinically infected cattle. Despite the evident theilericidal effect, BPQ showed only a transient efficacy against *T. orientalis* Ikeda and parasite relapse was observed in all treated animals. This outcome was observed regardless of the drug dose. Thus, the results indicate that BPQ did not completely remove the parasites from the infected calves, despite of its observable theilericidal effect. We administered a 6 mg/Kg dose in an attempt to increase the drug efficacy and duration in which the parasite remained undetectable. However, the dose increment from 2.5 to 6 mg/kg (2.4 times) did not necessarily reflect in a higher theilericidal effect.

Considering that only two and three animals treated with 2.5 mg/kg or 6 mg/Kg BPQ, respectively, were evaluated, no statistical analysis on the pharmacokinetics results was performed; however, results showed similar patterns as previously described for parvaquone and atovaquone (52). Notably, the dose proportionality parameters revealed that the serum concentrations increased less than proportional to the dose increment, which would explain why there was no evident increase in the pharmacologic effect when animals were dosed with 6 mg/kg. The reasons for the lack of dose proportionality of BPQ (2.5–6 mg/kg) are unclear but deserve to be studied in a larger pharmacokinetics study and question the therapeutic value of administering 6 mg/kg over 2.5 mg/kg of the drug. All these considered, efficacy of BPQ combined with additional anti-protozoal therapeutics and alternative dosage regimens for clearing *T. orientalis* Ikeda in sub-clinically infected cattle remains to be determined.

Hydronaphthoquine compounds target the cytochrome b (*cytb*) gene of apicomplexan protozoans (24, 53, 54). Numerous reports have shown that point mutations in the *cytb* gene are associated with parasite resistance to hydronaphthoquines (26, 27, 55–57). Considering the data presented here, it is reasonable to state that the parasite level fell below the limit of detection of the molecular assays used in this study; however, treatment was not 100% effective in clearing the infection, regardless of the drug dose. We propose at least three possibilities to explain these observations. First, there exists a minor subpopulation of resistant parasites. Second, drug levels were not high enough, or did not persist for enough time in the blood, to eliminate all the parasites. Third, a certain parasite subpopulation was inaccessible to the drug and therefore, not targeted due to its location or sequestration in the animal’s organs. Additional studies are needed to investigate these hypotheses. Intriguingly, a dose of 6 mg/kg showed no effect on the parasitemia in animals that had been previously treated with the BPQ label dose of 2.5 mg/kg. This lack of effect on parasitemia following the second treatment with a higher dose of BPQ suggests the emergence of a subpopulation of parasites that may have become resistant to the drug. Further mechanistic investigations are necessary to examine the potential *in vivo* selection of *T. orientalis* Ikeda following treatment with BPQ.

BPQ treatment had no effect on the total number of leukocytes, including lymphocytes, monocytes, and neutrophils, in peripheral blood, consistent with previous observations (46). Interestingly, in the present study, calves treated with either 2.5 mg/kg label dose or 6 mg/kg of BPQ showed a significant decrease in BUN two weeks after treatment. Even though the effect on BUN was reversible and occurred without alterations in ALT, ALP and GGT, a potential liver impairment associated with BPQ treatment may be considered, as previously described following atovaquone administration in humans (58). Alternatively, the decrease in BUN may have been caused by other factors than the drug treatment, such as alterations of dietary nitrogen status (59); however, no changes in the diet of the animals occurred throughout the experiment. Future studies on BPQ, alone or combined with other compounds, may take into consideration the potential development of liver damage, in addition to previously demonstrated long-term detection of residues of the drug in treated animals (29).

Collectively, considering the present data and the economic impact that *T. orientalis* Ikeda can have on the cattle industry, future investigations are necessary to evaluate novel drugs and regimens to treat and eliminate the parasite. Such therapeutics, combined with vaccines and diagnostic assays, are urgently needed to design integrated strategies to control and eradicate *T. orientalis* Ikeda from endemic and at-risk, emerging areas.

## 5 Conclusion

Here we demonstrated that BPQ is effective at rapidly reducing parasite load of the *T. orientalis* Ikeda, US isolate, to undetectable levels in peripheral blood, shortly after treatment, with the molecular assays used in this study. However, BPQ, as a monotherapeutic, was not effective in clearing the parasite in sub-clinically, asymptomatic infected cattle. The drug was used at two different doses, the label dose of 2.5 mg/kg and 6 mg/kg, and despite of its evident effect on decreasing parasitemia, parasite relapse was observed in all treated animals.

## Data availability statement

The original contributions presented in the study are included in the article/Supplementary material, further inquiries can be directed to the corresponding author.

## Ethics statement

The animal study was approved by Washington State University Institutional Animal Care and Use Committee. The study was conducted in accordance with the local legislation and institutional requirements.

## Author contributions

RB: Conceptualization, Data curation, Formal analysis, Funding acquisition, Investigation, Methodology, Project administration, Resources, Supervision, Validation, Visualization, Writing – original draft, Writing – review & editing. AH: Methodology, Writing – review & editing. CO: Formal analysis, Investigation, Methodology, Visualization, Writing – review & editing. DH: Conceptualization, Formal analysis, Investigation, Methodology, Visualization, Writing – review & editing. NV: Conceptualization, Formal analysis, Investigation, Methodology, Validation, Visualization, Writing – review & editing. JL: Investigation, Methodology, Writing – review & editing. LF: Conceptualization, Data curation, Formal analysis, Funding acquisition, Investigation, Methodology, Project administration, Resources, Supervision, Validation, Visualization, Writing – review & editing.
